# Supplementary material for: Viral replication and interferon responses in bronchial epithelia is enhanced by Th17 cells
Source: bioRxiv. 2025 Oct 12:2025.10.10.681711. Preprint. [Version 1] doi: 10.1101/2025.10.10.681711 (PMC12668410; doi:10.1101/2025.10.10.681711)
Supplement: Supplement 2 [file NIHPP2025.10.10.681711v1-supplement-2.pdf]

bioRxiv preprint doi: https://doi.org/10.1101/2025.10.10.681711; this version posted October 12, 2025. The copyright holder for this preprint (which was not certified by peer review) is the author/funder, who has granted bioRxiv a license to display the preprint in perpetuity. It is made available under aCC-BY 4.0 International license.

Supplemental Figures

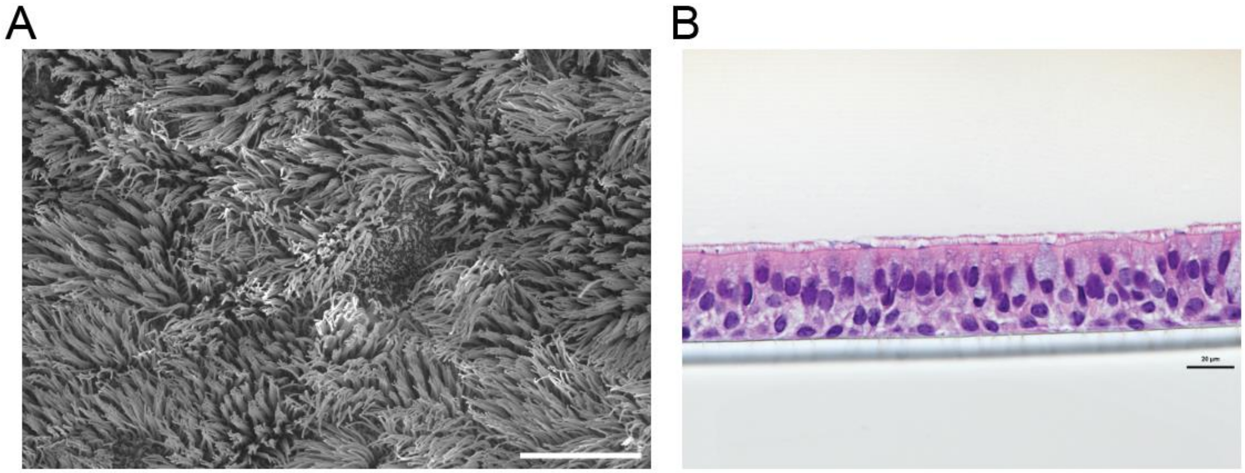

Figure E1. A. Scanning electron microscopy of a representative mature BEC culture showing ciliated apical layer. Scale bar 10 microns. **B**. H&E staining of a representative mature BEC culture with pseudostratified epithelium. Scale bar 25 microns.
